# Extension of shelf life of Nile tilapia (*Oreochromis niloticus*) fillets using seaweed extracts during refrigerated storage

**DOI:** 10.1002/fsn3.3673

**Published:** 2023-09-07

**Authors:** Jaki Shahrier, Golam Rasul, Faria Afrin, Rabiul Islam, A. K. M. Azad Shah

**Affiliations:** ^1^ Department of Fisheries Technology Bangabandhu Sheikh Mujibur Rahman Agricultural University Gazipur Bangladesh; ^2^ Department of Aquaculture Bangabandhu Sheikh Mujibur Rahman Agricultural University Gazipur Bangladesh

**Keywords:** Nile tilapia, quality, refrigeration storage, seaweed extracts, sensory evaluation, shelf life

## Abstract

The effects of seaweed (*Padina tetrastromatica*, *Sargassum natans*, and *Sargassum fluitans*) ethanolic extracts on the quality and shelf life extension of Nile tilapia (*Oreochromis niloticus*) fillets were investigated during refrigerated storage for 20 days. Each of the seaweed ethanolic extracts solution (2%, w/v) was used for dipping the fish fillets for 10 min at 4°C. The control and seaweed extract‐treated fillets were stored at 4 ± 1°C in air‐tight polyethylene bags, and chemical, bacteriological, and sensory evaluation were performed at every 4 days' intervals. During the storage period, *P*. *tetrastromatica* extract significantly (*p* < .05) reduced the increment of pH, peroxide value, thiobarbituric acid reactive substances, and total volatile basic nitrogen values in Nile tilapia fillets compared to other seaweed extracts‐treated and untreated fillets. The maximal total viable count of control, *P. tetrastromatica*, *S. natans*, and *S. fluitans* extracts‐treated fillets was 6.53, 7.11, 6.75, and 7.10 log CFU/g at the 8th, 20th, 12th, and 16th days of storage, respectively. The total psychrotrophic count of control and seaweed extracts‐treated fillets was also significantly increased (*p* < .05) throughout the storage period. The *P. tetrastromatica* extracts‐treated fillets showed better sensory characteristics than other seaweed extracts‐treated and control fillets. Results of this study suggest that ethanolic extracts (2%, w/v) of *P*. *tetrastromatica* extend the shelf life for 12 days longer than the control fillets in refrigerated conditions.

## Graphical abstract

During the storage period, *P. tetrastromatica* extract significantly (*p* < .05) reduced the increment of pH, peroxide value, thiobarbituric acid reactive substances, and total volatile basic nitrogen values in Nile tilapia fillets compared to other seaweed extracts‐treated and untreated fillets. The total viable count and total psychrotrophic count of control and seaweed extracts‐treated fillets were significantly increased (*p* < .05) throughout the storage period. Results of this study suggest that ethanolic extracts (2%, w/v) of *P. tetrastromatica* extend the shelf life for 12 days longer than the control fillets in refrigerated conditions.

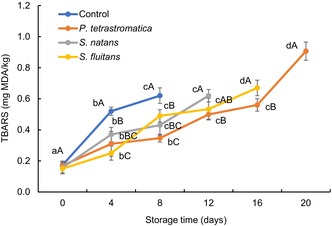

## 1 INTRODUCTION

Nile tilapia (*Oreochromis niloticus*) is the most important freshwater fish species contributing to an increase in global production and consumption of fish from aquaculture systems. In 2019–2020, the total fish production of Bangladesh was 4.503 million metric tons (MT); among them, tilapia's production was 371,263 MT (DoF, 2020). Tilapia is considered an ideal species for aquaculture, and it has earned the title “aquatic chicken.” In recent years, consumers have wanted to buy processed fish fillets because of their busy schedules. However, there are no such fishery products available in the markets or super shops in Bangladesh. Therefore, it is important to develop a safe and cost‐effective method for the preservation of value‐added fishery products to meet the huge consumer demand.

Fish is a highly perishable food item due to its rapid endogenous enzymatic and microbial activity in the postmortem stage, resulting in the production of undesirable metabolites that limit shelf life and cause loss of quality (Tavares et al., 2021; Uddin et al., 2017). To prevent the spoilage of fish, different methods such as refrigeration, chilling, icing, etc. are employed by lowering the temperature, and synthetic phenolic compounds are also used as antioxidants and antimicrobial agents to increase the shelf life and quality of fish or seafood (Brewer, 2011; Mei et al., 2019). However, the use of synthetic compounds for preserving food items is becoming limited with increasing concerns about food safety and health issues (Olatunde & Benjakul, 2018). Due to the potential toxicity of synthetic food additives, many studies have focused on natural phytochemicals, such as plant‐derived essential oils and polyphenols as food preservatives (Huang et al., 2017; Lytou et al., 2018; Vijayan et al., 2022). Plant extracts have vast applications in improving the texture of fish muscle and extending the shelf life of fishery products (Farvin et al., 2012; Raeisi et al., 2017).

Conversely, seaweeds are emerging as a viable and plentiful source of natural antioxidants and different kinds of bioactive compounds having health‐promoting properties (Garcia et al., 2020; Rengasamy et al., 2020). A number of potent antioxidant and antimicrobial compounds, including phenolic acids, flavonoids (e.g., anthocyanidins, catechins, flavones, flavonols, flavononols, isoflavonoids, proanthocyanidins, and quercitin), lignins, tocopherols, bromophenols, fucoxanthin, phlorotannins, etc. have been isolated and identified from different types of seaweed (Cotas et al., 2020; Gomes et al., 2022). Among the different seaweeds, brown seaweeds are known for their excellent antioxidant properties in comparison to green and red seaweeds (Movahhedin et al., 2018). Previous studies reported that ethanolic extracts of *Sargassum natans* and *Sargassum fluitans* contain polysaccharides, catechic tannins, quercetin, genistein, polyterpene sterols, saponosides, and phenolic compounds, particularly phlorotannins that have potent antioxidant and antimicrobial properties (Quattara et al., 2021). Besides, ethanolic extracts of *P. tetrastromatica* have shown potent antioxidant, and antibacterial activity against *Staphylococcus aureus*, *Enterococcus faecalis*, and *Pseudomonas aeruginosa* (Afrin, Ahsan, et al., 2023). The major active substances in *P. tetrastromatica* were identified as luteolin, genistein, rosmarinic acid, hydroxyferulic acid, quercetin, salvianic acid, phlorotannins, laminarin, glycolipid, and fucoxanthin (Naveen et al., 2021). Moreover, bioactive potential macromolecules (fucoidan or sulfated polysaccharide) of brown seaweeds showed strong antibacterial activity against various bacterial pathogens such as *Vibrio* spp., *Staphylococcus* sp., *Escherichia coli*, *Aeromonas hydrophila*, *Enterobacter* sp., *P. aeruginosa*, etc. (Palanisamy et al., 2017). Thus, the use of seaweed extract is an effective way to prevent lipid oxidation and retard microbial growth in fish or fishery products to maintain their nutritional quality and prolong their shelf life.

Many studies have shown the preservative effect of algae extracts on seafood such as chilled cod muscle (Wang et al., 2009), chilled megrim (Miranda et al., 2016), Pacific white shrimp (Li et al., 2017), shrimp (Balti et al., 2020), rainbow trout (Saez et al., 2021) etc. Husni and Wijaya (2013) also reported that ethanolic extract (2%, w/v) of *Gracilaria* sp. reduced the increasing rate of TVB‐N, TBARS, and TVC of red tilapia fillet with increasing storage time and also exhibits better sensory scores during refrigeration storage at 6°C. It has been reported that aqueous extract (2%, w/v) of *Padina tetrastromatica* effectively retards the spoilage rate and extends the shelf life of refrigerated *Pangasius* fillet (Deepitha et al., 2021). However, the application of *P*. *tetrastromatica*, *S*. *natans*, and *S*. *fluitans* extracts as natural preservatives has not been studied in Nile tilapia fillets. Therefore, this study aimed to evaluate the effects of seaweed extracts on the quality and shelf life of Nile tilapia fillets during refrigerated (4 ± 1°C) storage.

## 2 MATERIALS AND METHODS

### 2.1 Preparation of fish fillets

Live Nile tilapia (*O. niloticus*) (average weight of 650 ± 45 g) were collected from a fish farm located at Kaliakoir, Gazipur district of Bangladesh, and immediately slaughtered by dipping in ice‐cold water (hypothermia). The fish were kept in flack ice and carried to the Fish Processing Laboratory at Bangabandhu Sheikh Mujibur Rahman Agricultural University (BSMRAU), Gazipur. The fish was beheaded, gutted, washed, and filleted into two pieces using sterilized sharp knives. The fillets were cut into small pieces (approximately 5 cm × 6 cm × 3 cm), and the average weight of each piece was 45.4 ± 4.1 g.

### 2.2 Collection and preparation of seaweed extract

Three species of brown algae (*P. tetrastromatica*, *S. natans*, and *S. fluitans*) were collected from Saint Martin's Island, Cox's Bazar, Bangladesh. Seaweeds were transported to the laboratory and washed thoroughly with tap water and left to dry at room temperature (28–30°C) for 7 days (Moubayed et al., 2017). Dried seaweeds were cut into small pieces and ground into powder using a blender. The powdered seaweeds were packed in plastic bags and stored at −26°C until analysis.

Seaweeds were extracted using ethanol (purity > 99.8%) following the method described by Raeisi et al. (2016) with slight modifications. Powdered seaweeds (100 g) were soaked with 1000 mL of ethanol in a reagent bottle separately and gently mixed by shaking and left for 72 h at room temperature (26–29°C). The liquid phase was filtered using Whatman No. 1 filter paper and stored at 4°C. After filtration, the seaweeds were soaked in ethanol again and left for 72 h at room temperature. The liquid phase was filtered again and combined with the first extract and concentrated using a rotary evaporator (Stone, Staffordshire, ST15 0SA, UK). The crude extracts obtained were weighed to determine extracts yield and stored in dark at 4°C for further use.

### 2.3 Coating application and storage

For coating application, each of the seaweed extracts was dissolved into distilled water at a concentration of 2% (w/v). Fish fillets were randomly assigned into four treatment groups consisting of the first group was dipped into sterilized distilled water (control); the second group was dipped into *P. tetrastromatica* extract solution; the third group was dipped into *S. natans* extract solution; and the fourth group was dipped into *S. fluitans* extract solution. The duration of the dipping treatment was 10 min at 4°C. Then the fish fillets were drained on a pre‐sterilized metal net and air dried for 5 min in order to obtain an edible coating on the fish fillet (Dulal et al., 2023). All the treated fish fillets were separately packed in airtight polyethylene zipper bags and stored at 4 ± 1°C for 20 days in a refrigerator. Sampling was done at 4 days' intervals until apparent decomposition and chemical analysis were also performed accordingly. The samples were analyzed for chemical, bacterial, and sensory evaluation at each sampling to determine the quality and shelf life of the fish fillets.

### 2.4 Chemical analyses

The proximate composition (moisture, crude protein, crude lipid, and ash content) of fish fillets was analyzed according to the standard procedure given in Association of Official Analytical Chemists (AOAC, 2005). The pH value was directly measured using a pH meter following the method of Rasul, Kabir, et al. (2021). Total volatile basic nitrogen (TVB‐N) was determined according to the AOAC (2005) method and expressed as mg N/100 g muscle. The total lipid of fish fillets was extracted following the method described by Bligh and Dyer (1959). The peroxide value of the extracted lipid was determined according to AOAC (2005) method and expressed as meq O_2_/kg of lipid. The thiobarbituric acid reactive substance (TBARS) was measured according to the method described by Buege and Aust (1978), and the value was expressed as mg malondialdehyde (MDA)/kg of flesh.

### 2.5 Bacteriological analyses

The bacterial counts of fish fillets were determined according to the method of Afrin et al. (2021). Total viable count (TVC) and total psychrotrophic count (TPC) were measured by the pour plate method using plate count agar (HiMedia). The plates were incubated at 37°C for 2 days for TVC, and 4°C for 10 days for TPC. The values were expressed as log colony forming units (CFU)/g of flesh.

### 2.6 Sensory evaluation

The sensory evaluation of fish fillets was done following the method of Ojagh et al. (2010). Raw fillets were assessed by fifteen trained assessors (ages between 23 and 35 years) from the Department of Fisheries Technology of BSMRAU. Sensory characteristics were evaluated using a 5‐point scale to assess: color (5, no discoloration; 1, extreme discoloration); odor (5, extremely acceptable; 1, extremely undesirable/off‐odor); texture (5, extremely firm; 1, very soft); and overall acceptability (5, extremely acceptable; 1, extremely undesirable) of the samples. A sensory score above 4 implied that the fillets were acceptable for human consumption.

### 2.7 Statistical analyses

All data are expressed as the mean ± standard deviation. All experiments were performed in triplicate based on a completely randomized design. The data were subjected to a two‐way analysis of variance (ANOVA) followed by a post hoc test using Duncan's multiple range test to identify the differences among the means (*p* < .05) (SAS, 2003, SAS Institute).

## 3 RESULTS AND DISCUSSION

### 3.1 Proximate composition

The proximate composition of fresh Nile tilapia fillet was 71.73% moisture, 23.01% crude protein, 2.92% crude lipid, and 1.84% ash on a fresh weight basis. In another study, Alsaggaf et al. (2017) found a comparatively higher amount of moisture (79.6 ± 0.30%) and relatively lower amount of crude protein (17.56 ± 0.71%), crude lipid (1.81 ± 0.05%), and ash (0.78 ± 0.03) in fresh Nile tilapia fillets. Jadhav and Anal (2018) also reported that the moisture, crude protein, crude lipid, and ash content of whole Nile tilapia were 79.09 ± 0.23%, 16.30 ± 0.14%, 1.74 ± 0.10%, and 1.74 ± 0.10%, respectively. Generally, variations in the proximate composition of fish flesh can be correlated with their nutrition, size of fish, gender, habitat, feeding habit, time of catching, spawning cycle, and other extrinsic factors (Rasul, Jahan, et al., 2021). Nile tilapia is a farmed fish; therefore, its body composition, especially protein and lipid content may vary depending on which feed they were fed and water quality in the culture area.

### 3.2 pH value

**FIGURE 1:**
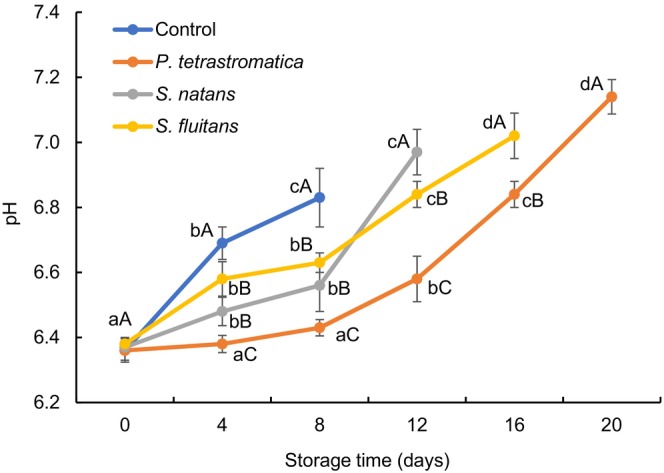
Changes in pH values of Nile tilapia fillets during refrigerated storage. The error bars represent means ± SD of triplicates. ^a–d^Small letters in each line indicate significant (*p* < .05) differences of means within the storage time. ^A–C^Capital letters indicate significant (*p* < .05) differences of means within the treatments.

The pH value was assessed as a crucial factor to determine the fish meat quality, which might interfere with the solubility activities of antioxidants. The pH values at day zero of all the treatments varied from 6.36 to 6.38, which indicates that the fish was fresh (Figure 1). The pH values were increased significantly (*p* < .05) in various amounts during refrigerated storage and reached to 6.83, 7.14, 6.97 and 7.02 at 8th, 20th, 12th, and 16th days for control, *P. tetrastromatica*, *S. natans*, and *S. fluitans*, respectively. An increment of pH was comparatively slower in seaweed extracts‐treated fillets as compared to control due to the presence of fucoxanthin and bromophenols in seaweeds that have antimicrobial properties, which eventually reduced the accumulation of alkaline compounds (Gomes et al., 2022). However, the subsequent increase of pH value may be due to the formation of volatile basic nitrogenous compounds, such as trimethylamine and ammonia that resulted from either microbial or endogenous enzymatic activities (Duman & Ozpolat, 2015). Similar results were also reported by Khalafalla et al. (2015), who found that the pH of refrigerated Nile tilapia fillets was gradually increased with storage time. Li et al. (2012) found that the use of rosemary (1.5%) and tea polyphenols (0.2%) extract has shown clear demarcation in lowering pH values for treated samples compared to those of control sample in crucian carp (*Carassius carassius*).

### 3.3 Peroxide value

**FIGURE 2:**
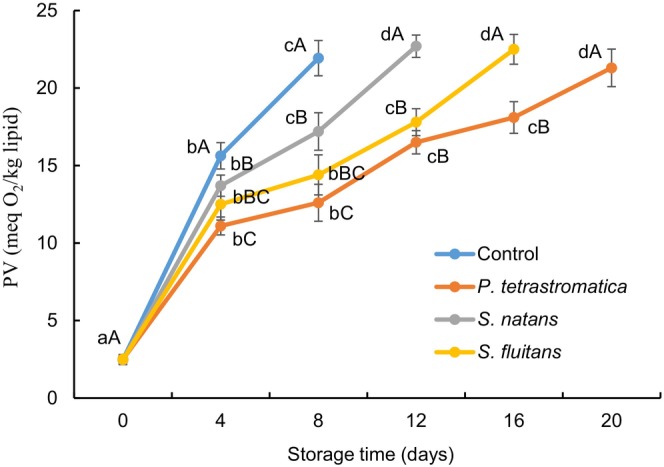
Changes in peroxide value (PV) of Nile tilapia fillets during refrigerated storage. The error bars represent means ± SD of triplicates. ^a–d^Small letters in each line indicate significant (*p* < .05) differences of means within the storage time. ^A–C^Capital letters indicate significant (*p* < .05) differences of means within the treatments.

Peroxide value (PV) is an indispensable index in determining primary lipid oxidation, which reduces the shelf life of fishery products. The initial PV values of Nile tilapia fillets ranged from 2.46 to 2.49 meq O_2_/kg lipid (Figure 2). During refrigerated storage, the PV of all the samples were increased significantly (*p* < .05) and the rate of increment was comparatively slower in seaweed extracts‐treated fillets than control fillets. The PV for control (15.63 meq O_2_/kg lipid), *P*. *tetrastromatica* (18.1 meq O_2_/kg lipid), *S*. *natans* (17.2 meq O_2_/kg lipid) and *S*. *fluitans* (17.8 meq O_2_/kg lipid) treated fillets was within acceptable range (PV < 20 meq O_2_/kg lipid; Afrin, Islam, et al., 2023) at 4th, 16th, 8th and 12th days of storage period, respectively. The control fillets showed a rapid increase in PV compared to seaweed extracts‐treated fillets during the storage time. It is because, seaweed contains several polyphenolic compounds, in particular phlorotannins, which inhibit the lipid peroxidation (Taniguchi et al., 2022). It has been reported that the PV of the control rainbow trout exceeded the acceptable value at 6th day, while it took 9 days for 1.5% shallot and 1.5% ajwain treated fillets during refrigerated storage (Raeisi et al., 2016). Similarly, Takyar et al. (2019) found that 0.1% *Chlorella vulgaris* and *Spirulina platensis* extracts were highly effective in delaying the production of PV during refrigerated storage of rainbow trout.

### 3.4 Thiobarbituric acid reactive substances

**FIGURE 3:**
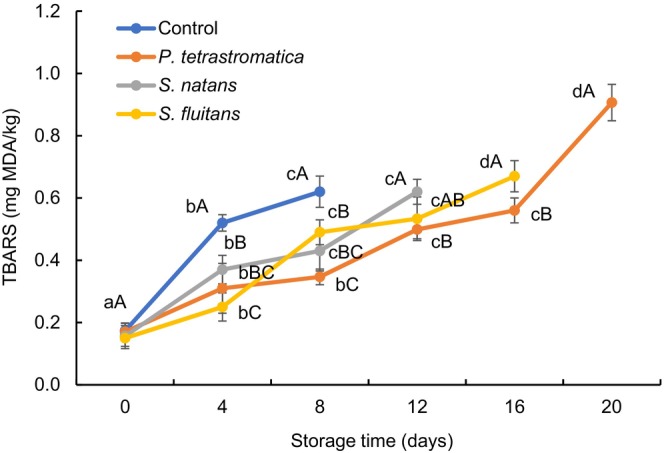
Changes in thiobarbituric acid reactive substances (TBARS) of Nile tilapia fillets during refrigerated storage. The error bars represent means ± SD of triplicates. ^a–d^ Small letters in each line indicate significant (*p* < .05) differences of means within the storage time. ^A–C^Capital letters indicate significant (*p* < .05) differences of means within the treatments.

The Thiobarbituric acid reactive substances (TBARS) value is considered an index of secondary lipid oxidation products by assessing MDA content. The TBARS values at day zero of all the treatments ranged between 0.15 and 0.17 mg MDA/kg fish tissue (Figure 3). The TBARS values of control and seaweed extracts‐treated fillets were significantly (*p* < .05) increased at different degrees and the values for control (0.62 mg MDA/kg), *P*. *tetrastromatica* (0.91 mg MDA/kg), *S*. *natans* (0.62 mg MDA/kg), and *S*. *fluitans* (0.67 mg MDA/kg) extracts were observed at 8th, 20th, 12th, and 16th days, respectively. The TBARS values observed in this study were within the acceptable limit (1–2 mg MDA/kg for fresh fish) (Afrin, Islam, et al., 2023). The TBARS values of seaweed extracts‐treated fillets were significantly (*p* < .05) lower, and the rate of increment was comparatively slower than that of control due to the presence of several antioxidants (viz. fucoxanthin, phlorotannins, and tocopherols) in brown seaweeds, which retarded the formation of volatile lipid oxidation products in the fish fillets (Airanthi et al., 2011). In this study, significantly the lowest TBARS value was found in the fillets treated with *P*. *tetrastromatica* extracts (2.0%), which agrees with the results of Deepitha et al. (2021), who observed that 2% *P. tetrastromatica* extract reduces the TBARS value throughout the chilled storage of *Pangasius* fillet. Moreover, an ethanolic extract of *P. fucoides* inhibits lipid oxidation and extends the shelf life of mackerel mince during chilled storage (Babakhani et al., 2016).

### 3.5 Total volatile basic‐nitrogen

**FIGURE 4:**
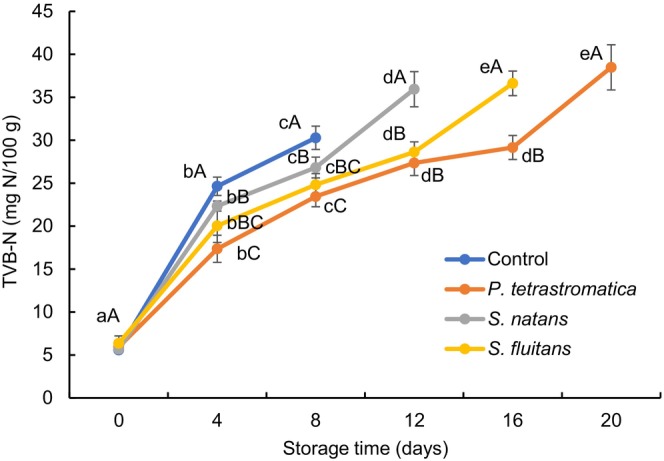
Changes in total volatile basic‐nitrogen (TVB‐N) values of Nile tilapia fillets during refrigerated storage. The error bars represent means ± SD of triplicates. ^a–e^Small letters in each line indicate significant (*p* < .05) differences of means within the storage time. ^A–C^Capital letters indicate significant (*p* < .05) differences of means within the treatments.

The total volatile basic‐nitrogen (TVB‐N) is generally composed of primary, secondary, and tertiary amines and ammonia. It increases with the activity of endogenous enzymes and microbial activity and gradually deteriorates the quality of fish (Rasul et al., 2018). The initial TVB‐N values of Nile tilapia fillets ranged from 5.59 to 6.37 mg N/100 g (Figure 4). The TVB‐N values were reached to 24.64, 29.15, 26.82, and 28.62 mg N/100 g at 4th, 16th, 8th, and 12th day of storage, respectively, for control, *P*. *tetrastromatica*, *S*. *natans*, and *S*. *fluitans* treated fillets, which was lower than the maximum acceptable level of TVB‐N value (TVB‐N < 30 mg N/100 g) for fishery products (Ocano‐Higuera et al., 2011). More or less similar TVB‐N values were observed when rosemary and sage tea extracts were used in vacuum packed refrigerated sardine fillets (Kenar et al., 2010) and alginate extract in refrigerated Japanese sea bass fillets (Cai et al., 2015). During refrigerated storage, the TVB‐N values were increased significantly (*p* < .05) in all the samples with the increasing of storage time. However, comparatively lower TVB‐N content was observed in seaweed extracts‐treated fillets that can be attributed to the antibacterial properties of fucoxanthin and phenolic compounds, particularly phlorotannins, which exhibited antibacterial activity through interaction with components of the bacterial system, ultimately leading to cell lysis (Gomes et al., 2022).

### 3.6 Bacteriological analyses

#### 3.6.1 Total viable count

**FIGURE 5:**
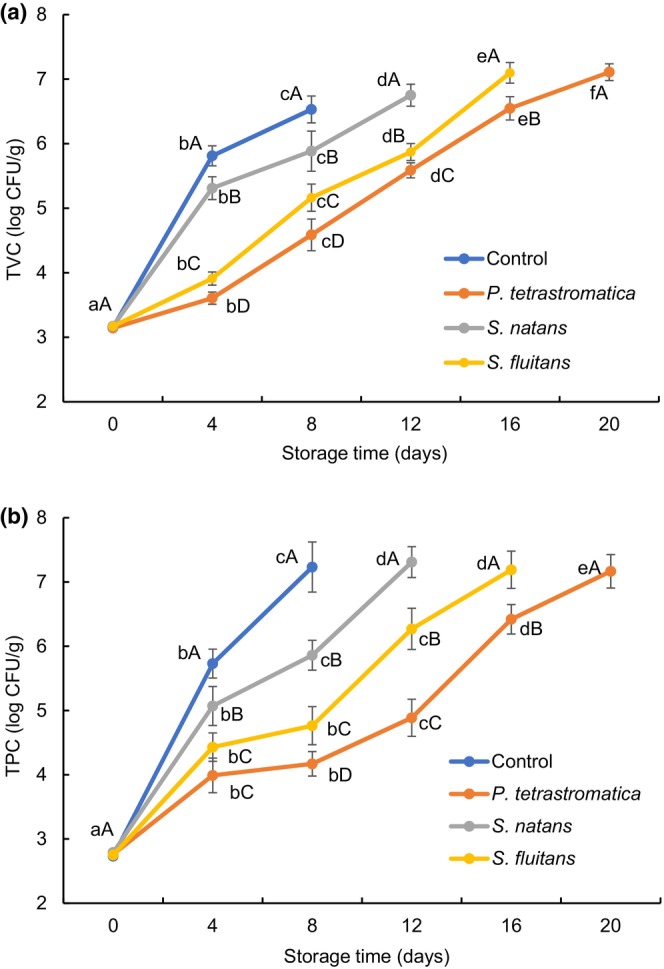
Changes in (a) total viable count (TVC) and (b) total psychrotrophic count (TPC) of Nile tilapia fillets during refrigerated storage. The error bars represent means ± SD of triplicates. ^a–f^Small letters in each line indicate significant (*p* < .05) differences of means within the storage time. ^A–D^Capital letters indicate significant (*p* < .05) differences of means within the treatment.

The total viable count is an important quality index for fish and fishery products. The initial TVC of Nile tilapia fillets at zero day ranged from 3.14 to 3.17 log CFU/g (Figure 5a). According to the recommended permissible limits (7 log CFU/g fish muscle) for fish by ICMSF (1986), the initial TVC of Nile tilapia indicates that the fish fillets are of good quality and fresh. The maximal TVC for control (6.53 log CFU/g), *P*. *tetrastromatica* (7.11 log CFU/g), *S*. *natans* (6.75 log CFU/g), and *S*. *fluitans* (7.10 log CFU/g) extracts‐treated fillets was found at the 8th, 20th, 12th, and 16th days of storage period, respectively. The TVC of all the treated and control fillets was significantly increased (*p* < .05) with the increasing of storage time. However, *P. tetrastromatica* and *S*. *fluitans* exhibited higher efficacy in lowering the bacterial activity and spoilage process. These results suggest that the presence of phenolic compounds in the seaweed extracts can be a reason for lesser extent of TVC in the seaweed treated fillets. The possible mechanism for disrupting the microbial cell wall with the benzene ring structure of phenolic compounds and the hydroxyl functional group, which easily penetrate the microbial cell and cross link with the enzymes resulting in cell death (Khalafalla et al., 2015). It has been reported that the ethanolic extract of *P. tetrastromatica* exhibited significantly (*p* < .05) higher antioxidant and antimicrobial activity due to the presence of the highest total phenolic content (Afrin, Ahsan, et al., 2023). Deepitha et al. (2021) found that *Padina* aqueous extract (2%) effectively reduced TVC and meat discoloration in *Pangasius* fillets up to 20 days of refrigerated storage.

#### 3.6.2 Total psychrotrophic count

The changes in total psychrotrophic count (TPC) of Nile tilapia fillets during refrigerated storage are shown in Figure 5b. The initial TPC in Nile tilapia fillets ranged between 2.73 and 2.79 log CFU/g flesh, which indicates that a premium quality fish was used in this study. The TPC was increased significantly (*p* < .05) with the increase of storage time, and the values were exceeded the permissible limit (7 log CFU/g) at the 8th, 20th, 12th, and 16th days for the control, *P*. *tetrastromatica*, *S*. *natans*, and *S*. *fluitans* extracts‐treated fillets, respectively. However, TPC counts were comparatively lower in seaweed extracts‐treated fillets than those of control fillets, which might be due to the presence of polyphenolic compounds in seaweed extracts. It is presumed that polyphenolic compounds have antibacterial activity through interaction with bacterial membrane penetrability, enzymatic deactivation, binding to surface membranes and surface adhesive molecules etc. (Silva et al., 2020). Shao et al. (2022) also found that polyphenol treatment effectively inhibits the growth and reproduction of the psychrophilic bacteria viz. *Pseudomonas*, *Acinetobacter* and *Aeromonas* during partial freezing of tilapia fillets. More or less similar results were observed by Yazgan et al. (2020), they reported that the ethanolic extract of propolis was effective in inhibiting bacterial growth in sardine fillets during chilled storage (3 ± 1°C). It has been reported that psychrotrophic bacteria are the vital group of microbes accountable for the decomposition of aerobically preserved fish at refrigerated condition (4 ± 2°C) (Durmus, 2020).

### 3.7 Sensory evaluation

**FIGURE 6:**
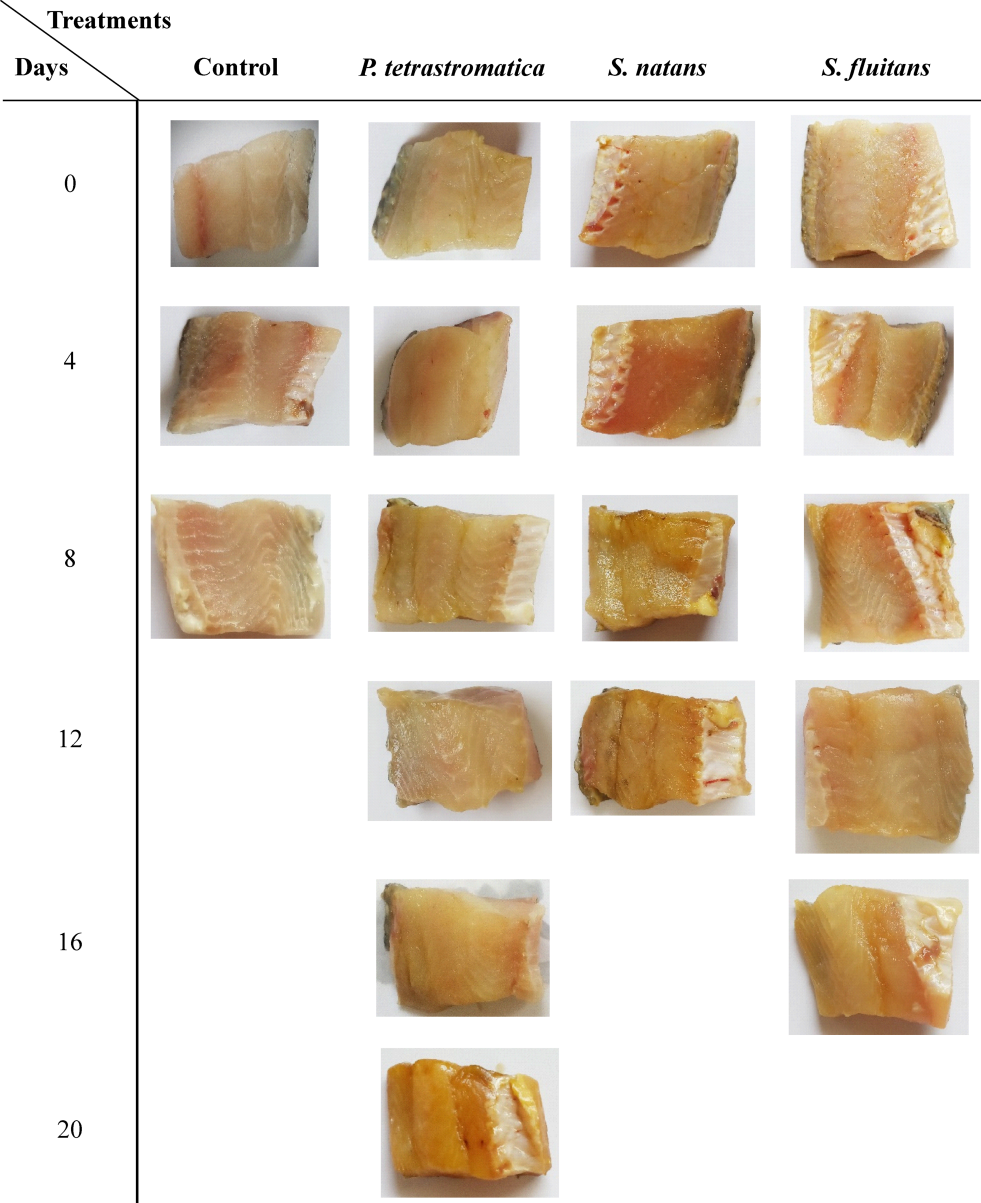
Visual appearance of Nile tilapia fillets during refrigerated storage.

To determine the quality and shelf life of Nile tilapia fillets, trained panel members were assessed the sensory attributes such as color, odor, texture and overall acceptability. Sensory evaluation results revealed that the control, *P*. *tetrastromatica*, *S*. *natans*, and *S*. *fluitans* treated samples were found unacceptable at the 8th, 20th, 12th, and 16th days, respectively (Table 1). However, all the sensory attributes were deteriorated slowly in *P*. *tetrastromatica* extracts‐treated fillets than other seaweed extracts‐treated fillets as well as control fillets. During the dipping treatment, color was developed on the seaweed extracts‐treated fillets and it was changed with the increase of storage time (Figure 6). This might be due to the presence of chlorophyll and phycocyanin in the seaweeds. Moreover, the color developed on the fillets had no adverse effect in the sensory attributes. Shao et al. (2022) reported that polyphenols such as procyanidin, carnosic acid, resveratrol, and quercetin mostly retained the color and flavor quality of tilapia fillets during partial freezing. In contrast, discoloration of muscle, loss of muscle elasticity and development of off odor were gradually increased with the increase of storage time in all the treated and untreated fillets. Sensory evaluation results also correlate with the chemical and microbial data, which is similar to the findings of other researchers (Raeisi et al., 2016; Rajasekar et al., 2020). Moreover, a comparatively better sensory score and extended shelf life were found in refrigerated Bighead carp (*Aristichthys nobilis*) fillets when treated with ethanolic and aqueous pomegranate peel extracts (Zhuang et al., 2019).

**TABLE 1:** Changes in sensory attributes of Nile tilapia fillets during refrigerated storage.

Storage time (days)	Control	*Padina tetrastromatica*	*Sargassum natans*	*Sargassum fluitans*
Color				
0	4.98 ± 0.03aA	4.96 ± 0.05aA	4.96 ± 0.05aA	4.95 ± 0.09aA
4	4.56 ± 0.11bB	4.92 ± 0.08aA	4.91 ± 0.10aA	4.90 ± 0.11aA
8	3.78 ± 0.08cC	4.89 ± 0.09aA	4.29 ± 0.22bB	4.88 ± 0.11aA
12		4.38 ± 0.18bA	3.68 ± 0.19cB	4.33 ± 0.11bA
16		4.18 ± 0.15cA		3.74 ± 0.35cB
20		3.64 ± 0.17dA		
Odor				
0	4.98 ± 0.03aA	4.96 ± 0.05aA	4.98 ± 0.03aA	4.95 ± 0.09aA
4	4.46 ± 0.19bB	4.90 ± 0.12aA	4.82 ± 0.13aA	4.86 ± 0.09abA
8	3.24 ± 0.15cC	4.81 ± 0.14aA	4.13 ± 0.11bB	4.66 ± 0.15bA
12		4.39 ± 0.11bA	3.52 ± 0.19cB	4.20 ± 0.16cA
16		4.25 ± 0.14bA		3.45 ± 0.26 dB
20		3.70 ± 0.16cA		
Texture				
0	4.98 ± 0.03aA	4.96 ± 0.05aA	4.98 ± 0.04aA	4.95 ± 0.09aA
4	4.52 ± 0.10bB	4.91 ± 0.15abA	4.85 ± 0.16aA	4.89 ± 0.10abA
8	3.59 ± 0.11cC	4.79 ± 0.07bA	4.26 ± 0.25bB	4.74 ± 0.13bA
12		4.50 ± 0.14cA	3.74 ± 0.11cC	4.12 ± 0.13cB
16		4.12 ± 0.13dA		3.58 ± 0.16 dB
20		3.66 ± 0.11eA		
Overall acceptability				
0	4.98 ± 0.03aA	4.96 ± 0.05aA	4.97 ± 0.04aA	4.95 ± 0.09aA
4	4.61 ± 0.21bB	4.91 ± 0.08aA	4.86 ± 0.13aAB	4.88 ± 0.11aA
8	3.36 ± 0.29cC	4.83 ± 0.16aA	4.23 ± 0.21bB	4.76 ± 0.11bA
12		4.42 ± 0.22bA	3.65 ± 0.26cC	4.22 ± 0.08cB
16		4.18 ± 0.13cA		3.59 ± 0.24 dB
20		3.67 ± 0.22dA		

*Note*: The values are expressed as mean ± standard deviation (*n* = 15). The small letters within the same column denote significant (*p* < .05) differences of means between storage times, while the capital letters within the same row denote significant (*p* < .05) differences of means between various treatments. The acceptable sensory score is above 4 for fresh fish (Ojagh et al., 2010).

## 4 CONCLUSIONS

The results of this study demonstrated that seaweed extracts effectively reduced the increment rate of pH, PV, TBARS, and TVB‐N values in Nile tilapia fillets compared to control fillets during refrigerated storage. Moreover, *P*. *tetrastromatica* extracts exhibited comparatively higher efficacy in inhibiting bacterial growth in fish fillets than other seaweed extracts‐treated and control fillets. The fish fillets treated with *P*. *tetrastromatica* extracts had acceptable sensory attributes up to the 16th day, while the control fillets were acceptable up to the 4th day of storage. Due to the presence of various hydrophilic compounds, the seaweed extracts retarded the bacterial growth as well as inhibited the formation of ammonia and other secondary lipid oxidation products, thus retaining the quality of the fish fillets for a longer period of time and providing good sensory attributes. Chemical, bacteriological, and sensory evaluation results revealed that *P*. *tetrastromatica* extracts (2%, w/v) were highly effective in retaining the quality and prolonging the shelf life of Nile tilapia fillets for 16 days during storage in refrigerated conditions. Therefore, ethanolic extracts of *P*. *tetrastromatica* can be used as natural preservative in the food industry for the preservation of fishery products.

## AUTHOR CONTRIBUTIONS

**Md. Jaki Shahrier:** Investigation (equal); methodology (equal); writing – original draft (equal). **Md. Golam Rasul:** Investigation (equal); methodology (equal); writing – original draft (equal). **Faria Afrin:** Investigation (equal); methodology (equal); writing – original draft (equal). **Md. Rabiul Islam:** Data curation (equal); validation (equal); writing – review and editing (equal). **A.K.M. Azad Shah:** Conceptualization (lead); data curation (lead); funding acquisition (lead); methodology (lead); project administration (lead); resources (lead); supervision (lead); validation (lead); writing – review and editing (lead).

## FUNDING INFORMATION

This work was financed by the Ministry of Education, Government of the People's Republic of Bangladesh (Grant No. LS2018716).

## CONFLICT OF INTEREST STATEMENT

The authors declare that there is no conflict of interest.

## ETHICS STATEMENT

The study was approved by the research ethics committee of Bangabandhu Sheikh Mujibur Rahman Agricultural University, Gazipur 1706, Bangladesh (Ref. No. FVMAS/AREC/2022/02).

## Data Availability

The data that support the findings of this study are available from the corresponding author upon reasonable request.
